# Cloning of the Herpes smplex virus Type 1 genome as an novel luciferase infectious bacterial artificial chromosome

**Published:** 2011-01-10

**Authors:** You Li, Shuai Wang, Junji Xing, Hua Zhu, Chunfu Zheng

**Affiliations:** 1 Molecular Virology and Viral Immunology Research Group, State Key Laboratory of Virology, Wuhan Institute of Virology, Chinese Academy of Sciences, Wuhan 430071, PR China; 2 Department of Microbiology and Molecular Genetics, UMDNJ-New Jersey Medical School, Newark, NJ 07101, USA

## 

Herpes simplex virus type 1 (HSV-1) is a ubiquitous human pathogen of skin and mucous membranes which associates with the infections of the mucocutaneous membranes, brain, and internal organs of infected neonates. As a member of the human herpesvirus family, HSV-1 contains a large DNA genome, encoding 84 unique open reading frames (ORFs), but the majority of its function is still elusive. In the present study, the genome of HSV-1 F strain was cloned as a stable and infectious BAC without any deletions of the viral genes. The BAC backbone sequences flanked by loxP sites were inserted into the intergenic region between UL37 and UL38. Cotransfection of the recombinant virus with a Cre recombinase plasmid resulted in the excision of the BAC sequences. Additionally, a firefly luciferase cassette was inserted to generate a novel luciferase HSV-1 BAC. Importantly, the resulting recombinant HSV-1 BAC_Luc_ behaved indistinguishably from the wild-type virus in vero cells, and the resulting luciferase activity could be quantified in vitro expediently. The recombinant HSV-1 BAC_Luc_ will facilitate HSV-1 research and provide the opportunity to exploit the power of BAC technology for production of recombinant viral vaccines.

